# Supplementary material for: How Healthy Are Health-Related Behaviors in University Students: The HOLISTic Study
Source: Nutrients. 2021 Feb 19;13(2):675. doi: 10.3390/nu13020675 (PMC7926492; doi:10.3390/nu13020675)
Supplement: Supplementary file 1 [file nutrients-13-00675-s001.zip › nutrients-1067438-supplementary.pdf]

Table S1. Description of the sample included in the HOLISTIC study (Habits, orthorexia nervosa and lifestyle in university students).

Country	University (Ethical approval)	Course/study program (year of data collection)	Course year	Total N of students enrolled	Response rate (%)	Method of data collection
**Croatia**	University of Split* (Ethics Committee of the University of Split School of Medicine; Reg. No: 2181-198-03-04-18-0027)	Medicine (2018)	1	90	92.2	Paper-based survey
			2	87	83.9	
			3	96	83.3	
			4	90	85.6	
			5	73	76.7	
			6	90	75.6	
		Medicine (2019)	1	90	82.2	Paper-based survey
			5	80	90.0	
			6	69	97.1	
		Dental medicine (2018)	1	204	94.3	Online survey
			2	42	88.1	
			3	33	75.8	
			4	27	88.9	
			5	44	84.1	
			6	23	69.6	
		Pharmacy (2018)	1	30	86.7	Online survey
			2	28	96.4	
			3	29	75.9	
			4	31	93.6	
			5	31	83.9	
		Nursing, radiology technology, lab technology, midwifery (2019)	1-3	390	77.7	Paper-based survey

		Economy (2018)	1-3	374	40.6	
**Italy**	University of Pavia (local ethical approval not needed)	Medicine and surgery (2018-2019)	1	141	70.5	Paper-based survey
			2	47	24.6	
			3	77	32.6	
			4	85	11.3	
			5	8	4.3	
			6	26	7.2	
		Physiotherapy (2018)	7	11	33.3	
			8	19	44.2	
		Neurodevelopmental therapy (2018)	1	9	19.0	
		Orthotics and prosthetics (2018)	1	15	35	
		Nursing (2019)	1	99	47.6	
			2	133	67.7	
			3	63	36.1	
			-	Course year information was not reported by 52 students	na	
		Dentistry (2019)	1	19	63.3	
			2	10	20.0	
			3	15	88.2	
			4	14	77.8	
			5	6	42.9	
		Obstetrics (2019)	1	16	84.2	
			2	13	81.2	

			-	Course year information was not reported by 2 students	na	
			3	17	85.0	
	University of Foggia (local ethical approval not needed)	Medicine and surgery (2018-2019)	1-6	597	67.5	Online survey and paper-based survey
		Nursing studies (2018-2019)	1-3	337	62.6	
		Biomedical laboratory techniques (2018-2019)	1-4	62	82.3	
		Biomoleculars sciences and technology (2018-2019)	1-2	47	100.0	
		Dietetics (2018-2019)	1-4	51	100.0	
		Physiotherapy (2018-2019)	1-3	100	90.0	
		Sport sciences, physical exercise and human health (2018-2019)	1-5	235	80.4	
		Dentistry and dental implants (2018-2019)	1-6	214	100.0	
		Business administration (Bachelor degree) (2018-2019)	1-3	741	62.4	
		Economics (bachelor degree) (2018-2019)	1-3	479	63.3	
		Marketing management (2018-2019)	1-5	95	67.4	
		Economics and finance (2018-2019)	1-5	59	86.4	
		Business administration (master degree) (2018-2019)	1-5	201	93.5	
**Lebanon**	Beirut Arab University (IRB Approval: 2019H-0089-HS-R-0327)	Nutrition & Dietetics (2019-2020)	1	50	96.0	Paper-based survey
			2	26	65.4	
			3	44	84.1	
		Physical Therapy (2019)	1	68	80.9	

			2	37	64.9	
			3	23	43.5	
			4	53	11.3	
		Nursing (2019)	1	75	93.3	
			2	34	94.1	
			3	21	81.0	
		Medicine (2019)	1	135	25.2	
			2	117	23.1	
		Dentistry (2019)	2	87	27.6	
**Poland**	University of Wroclaw (Ethics Committee of the Faculty of Psychology in Katowice at the SWPS University of Social Sciences and Humanities; WKEB56/04/2019)	Medicine (2019)		82	85	Online survey
		Social Sciences (e.g. psychology, sociology) (2019)	4	101	92	
		Sciences (2019)	3	62	84	
**Romania**	University of Agricultural Sciences and Veterinary Medicine, Cluj-Napoca (local ethical approval not needed)	Food science (2020)	4	110	9	Online survey
		Environmental engineering (2020)	4	34	18	
		Veterinary medicine (2019-2020)	1	286	8	
			4	231	5	
			6	183	81	
		Engineering and management (2020)	4	19	42	
**Spain**	Universidad Pablo de Olavide, Sevilla (Ethics Committee of the Pablo de Olavide University; PEIBA	Human Nutrition & Dietetics (2020)	1	62	51.6	Paper-based survey
			2	64	76.5	
			3	56	82.1	
			4	62	100.0	

	reference number 20191118143720)	Sport Sciences (2020)	1	144	72.9	
			2	150	38.6	
			3	135	75.5	
			4	222	36.4	
**Turkey**	Koç University, Istanbul (Ethics Committee of the Koç University; Protocol No: 2018.242.IRB3.169)	Medicine and surgery (2018)	1	30	52.0	Paper-based survey
			2	25		
			3	28		
			4	18		
			5	24		
			6	17		
		Nursing (2018)	1	16	91.6	
			2	21		
			4	29		

Legend. *Coordinator of the study; na – not applicable.

Table S2. Questionnaires filled in by the students in the HOLISTIC study.

	General information	Dietary habits		Eating behaviour		Lifestyle and nutrition knowledge	Physical activity	Sleeping habits and screen-time	Perceived level of stress /quality of life
**Country (Language of courses)**	**Questions**	**Questions**	**MDSS** [20]	**ORTO-15** [22-25]	**EHQ** [21]	**Questions**	**IPAQ** [26,27] **/questions**	**Questions**	**PSS** [28,29] **/questions**
**Croatia (Croatian)**	Open-ended/ multiple-choice	Multiple-choice	FBT	FBT	FBT	Open-ended/ multiple-choice	IPAQ/ multiple-choice	Open-ended/ multiple-choice	FBT/ Open-ended/ multiple-choice
**Italy (Italian)**	Open-ended/ multiple-choice	Multiple-choice	FBT	ORTO-15	FBT	Open-ended/ multiple-choice	IPAQ/ multiple-choice	Open-ended/ multiple-choice	FBT/ Open-ended/ multiple-choice
**Lebanon (English)**	Open-ended/ multiple-choice	Multiple-choice	MDSS	ORTO-15	EHQ	Open-ended/ multiple-choice	IPAQ/ multiple-choice	Open-ended/ multiple-choice	PSS-10/ Open-ended/ multiple-choice
**Turkey (English)**	Open-ended/ multiple-choice	Multiple-choice	MDSS	ORTO-15	EHQ	Open-ended/ multiple-choice	IPAQ/ multiple-choice	Open-ended/ multiple-choice	PSS-10/ Open-ended/ multiple-choice
**Spain (Spanish)**	Open-ended/ multiple-choice	Multiple-choice	FBT	ORTO-11	FBT	Open-ended/ multiple-choice	IPAQ/ multiple-choice	Open-ended/ multiple-choice	PSS-14
**Poland (Polish)**	Open-ended/ multiple-choice	Multiple-choice	FBT	ORTO-15	FBT	Open-ended/ multiple-choice	IPAQ/ multiple-choice	Open-ended/ multiple-choice	FBT/ Open-ended/ multiple-choice

**Romania (English)**	Open-ended/ multiple-choice	Multiple-choice	MDSS	ORTO-15	EHQ	Open-ended/ multiple-choice	IPAQ/ multiple-choice	Open-ended/ multiple-choice	PSS-10/Open-ended/ multiple-choice

Legend. FBT - forward-back-translation [19].

Table S3. Post-hoc P values for between-countries comparison of the overall lifestyle characteristics of students, corresponding to the analysis presented in Table 1.

	**Post-hoc P¶**
Age *(years)* *median (IQR)*	<0.001[12]; 0.006[13]; <0.001[14];<0.001[15]; <0.001[16]; 0.162[17]; 0.026[18]; <0.001[23]; <0.001[24]; <0.001[25]; <0.001[26]; <0.001[27]; <0.001[28]; <0.001[34]; <0.001[35]; <0.001[36]; 0.304[37]; 0.735[38]; <0.001[45]; <0.001[46]; <0.001[47]; <0.001[48]; <0.001[56]; <0.001[57]; <0.001[58]; <0.001[67]; <0.001[68]; 0.265[78]
Female gender *n (%)*	<0.001[12]; <0.001[13]; <0.001[14];<0.131[15]; <0.194[16]; <0.001[17]; <0.001[18]; <0.001[23]; 0.001[24]; <0.001[25]; <0.001[26]; <0.001[27]; 0.078[28]; 0.096[34]; <0.001[35]; 0.207[36]; <0.001[37]; 0.048[38]; <0.001[45]; 0.022[46]; <0.001[47]; 0.548[48]; 0.032[56]; <0.001[57]; <0.001[58]; <0.001[67]; 0.011[68]; <0.001[78]
Grade point average *(percentile)* *median (IQR)*	0.097[12]; <0.001[13]; 0.007[14]; <0.001[15]; 0.014[16]; <0.001[17]; <0.001[18]; 0.931[23]; <0.001[24]; <0.001[25]; 0.072[26]; <0.001[27]; <0.001[28]; <0.001[34]; <0.001[35]; 0.051[36]; <0.001[37]; <0.001[38]; <0.001[45]; <0.001[46]; 0.006[47]; 0.001[48]; <0.001[56]; <0.001[57]; <0.001[58]; <0.001[67]; <0.001[68]; 0.004[78]
Smoking *n (%)* *yes* *ex-smokers* *never smoked*	<0.001[12]; <0.001[13]; 0.028[14]; 0.679[15]; <0.001[16]; <0.001[17]; 0.074[18]; 0.083[23]; <0.060[24]; <0.001[25]; <0.001[26]; <0.001[27]; 0.230[28]; 0.030[34]; <0.001[35]; <0.001[36]; <0.001[37]; 0.548[38]; 0.062[45]; 0.004[46]; <0.001[47]; 0.462[48]; 0.049[56]; <0.001[57]; 0.051[58]; <0.001[67]; 0.001[68]; 0.002[78]
BMI *(kg/m²)* *median (IQR)*	<0.001[12]; <0.001[13]; <0.001[14]; 0.004[15]; 0.094[16]; <0.001[17]; 0.862[18]; <0.001[23]; <0.001[24]; <0.001[25]; 0.013[26]; <0.001[27]; <0.001[28]; <0.001[34]; 0.541[35]; <0.001[36]; <0.001[37]; 0.001[38]; <0.001[45]; 0.002[46]; <0.001[47]; <0.001[48]; 0.002[56]; <0.001[57]; 0.037[58]; 0.584[67]; 0.292[68]; 0.051[78]
Self-rated health perception *median (IQR)*	<0.001[12]; <0.001[13]; <0.001[14]; 0.007[15]; <0.001[16]; 0.010[17]; <0.001[18]; 0.001[23]; <0.001[24]; <0.001[25]; 0.308[26]; 0.010[27]; <0.001[28]; 0.001[34]; <0.001[35]; 0.392[36]; <0.001[37]; 0.012[38]; 0.156[45]; 0.003[46]; <0.001[47]; 0.881[48]; <0.001[56]; <0.001[57]; 0.148[58]; 0.012[67]; 0.010[68]; <0.001[78]
Breakfast daily *n (%)*	<0.001[12]; <0.001[13]; <0.001[14]; <0.001[15]; 0.001[16]; <0.001[17]; <0.001[18]; 0.776[23]; <0.001[24]; <0.001[25]; <0.001[26]; 0.010[27]; <0.001[28]; 0.001[34]; <0.001[35]; <0.001[36]; <0.001[37]; <0.001[38]; <0.001[45]; 0.786[46]; <0.001[47]; 0.129[48]; <0.001[56]; 0.950[57]; <0.001[58]; <0.001[67]; 0.269[68]; <0.001[78]
ORTO-15 score *median (IQR)*	<0.001[12]; <0.001[13]; <0.001[14]; <0.001[15]; 0.569[16]; -[17]; 0.407[18]; <0.001[23]; <0.001[24]; <0.001[25]; 0.018[26]; -[27]; <0.001[28]; 0.359[34]; 0.002[35]; 0.001[36]; -[37]; 0.125[38]; 0.043[45]; 0.001[46]; -[47]; 0.052[48]; <0.001[56]; -[57]; 0.002[58]; -[67]; 0.292[68]; -[78]
MDSS score *median (IQR)*	<0.001[12;] <0.001[13]; 0.944[14]; 0.819[15]; <0.001[16]; <0.001[17]; 0.053[18]; <0.001[23]; <0.001[24]; <0.001[25]; 0.079[26]; <0.001[27]; <0.001[28]; <0.001[34]; <0.001[35]; <0.001[36]; 0.149[37]; <0.001[38]; 0.803[45]; <0.001[46]; <0.001[47]; 0.090[48]; <0.001[56]; <0.001[57]; 0.138[58]; <0.001[67]; <0.001[68]; <0.001[78]
Sleep duration on working days *(h)* *median (IQR)*	<0.001[12;] <0.001[13]; 0.020[14]; <0.001[15]; <0.001[16]; <0.001[17]; 0.002[18]; <0.001[23]; <0.001[24]; <0.001[25]; <0.001[26]; 0.001[27]; <0.001[28]; <0.001[34];<0.823[35]; 0.747[36]; 0.001[37]; <0.001[38]; <0.001[45]; <0.001[46]; <0.001[47]; <0.001[48]; 0882[56]; 0.024[57]; <0.001[58]; 0.082[67]; <0.001[68]; <0.001[78]

Sleep duration on non-working days *(h)* *median (IQR)*	<0.001[12]; <0.001[13]; <0.001[14]; 0.126[15]; <0.001[16]; 0.414[17]; 0.021[18]; <0.001[23]; <0.001[24]; <0.001[25]; <0.001[26]; <0.001[27]; <0.001[28]; <0.001[34]; <0.001[35]; <0.001[36]; <0.001[37]; 0.016[38]; 0.019[45]; 0.570[46]; <0.001[47]; <0.001[48]; 0.087[56]; 0.055[57]; 0.003[58]; <0.001[67]; <0.001[68]; 0.094[78]
Total METs per week† *median (IQR)*	<0.001[12]; 0.843[13]; <0.001[14]; 0.042[15]; 0.002[16]; <0.001[17]; <0.001[18]; <0.001[23]; <0.001[24]; <0.001[25]; 0.398[26]; <0.001[27]; <0.001[28]; <0.001[34]; 0.045[35]; 0.004[36]; <0.001[37]; <0.001[38]; 0.005[45]; <0.001[46]; <0.001[47]; 0.034[48]; <0.001[56]; <0.001[57]; <0.001[58]; <0.001[67]; <0.001[68]; <0.001[78]
Daily mobile use *(h)* *median (IQR)*	<0.001[12]; <0.001[13]; <0.001[14]; 0.432[15]; 0.678[16]; 0.514[17]; <0.001[18]; <0.001[23]; <0.001[24]; <0.001[25]; 0.002[26]; <0.001[27]; <0.001[28]; <0.001[34]; <0.001[35]; <0.001[36]; <0.001[37]; 0.363[38]; <0.001[45]; <0.001[46]; <0.001[47]; <0.001[48]; 0.337[56]; 0.759[57]; 0.001[58]; 0.372[67]; <0.001[68]; <0.001[78]
Daily study time *(h)* *median (IQR)*	<0.001[12]; <0.001[13]; 0.246[14]; <0.001[15]; <0.001[16]; <0.001[17]; 0.686[18]; <0.001[23]; <0.001[24]; <0.001[25]; <0.001[26]; <0.001[27]; <0.001[28]; <0.001[34]; <0.001[35]; <0.001[36]; <0.001[37]; <0.001[38]; <0.001[45]; <0.001[46]; <0.001[47]; 0.774[48]; 0.009[56]; 0.004[57]; <0.001[58]; 0.517[67]; 0.001[68]; <0.001[78]
PSS-10 score *median (IQR)*	<0.001[12]; 0.020[13]; <0.001[14]; 0.036[15]; 0.001[16]; <0.001[17]; <0.001[18]; 0.177[23]; <0.001[24]; 0.832[25]; 0.229[26]; <0.001[27]; <0.001[28]; <0.001[34]; 0.545[35]; 0.062[36]; <0.001[37]; <0.001[38]; 0.001[45]; 0.024[46]; <0.001[47]; 0.268[48]; 0.272[56]; <0.001[57]; <0.001[58]; <0.001[67]; 0.003[68]; <0.001[78]
Students asking for more education on lifestyle and health *n (%)*	<0.001[12]; <0.001[13]; 0.899[14]; -[15]; <0.001[16]; 0.863[17]; <0.001[18]; 0.113[23]; 0.001[24]; -[25]; 0.008[26]; <0.001[27]; 0.001[28]; <0.001[34]; -[35]; 0.091[36]; <0.001[37]; 0.018[38]; -[45]; <0.001[46]; 0.978[47]; <0.001[48]; -[56]; -[57]; -[58]; <0.001[67]; 0.566[68]; <0.001[78]

Legend. Man-Whitney U test was used as a post hoc test for numerical variables, and chi-square test was used as a post hoc test for categorical variables. A Bonferroni correction for post-hoc analysis was applied, yielding P value of ≤0.002 to be considered as statistically significant result.

Study sites correspond to numbers in superscript of the post-hoc P values: [1]Croatia, [2]Foggia, Italy, [3]Pavia, Italy, [4]Lebanon, [5]Poland, [6]Romania, [7]Spain, [8]Turkey. Hence, symbol [12] denotes comparison between Croatian and Foggia, symbol [13] denotes comparison between Croatia and Pavia, symbol [14] denotes comparison between Croatia and Lebanon, symbol [15] denotes comparison between Croatia and Poland, symbol [16] denotes comparison between Croatia and Romania, symbol [17] denotes comparison between Croatia and Spain, symbol [18] denotes comparison between Croatia and Turkey, symbol [23] denotes comparison between Foggia and Pavia, symbol [24] denotes comparison between Foggia and Lebanon, symbol [25] denotes comparison between Foggia and Poland, symbol [26] denotes comparison between Foggia and Romania, symbol [27] denotes comparison between Foggia and Spain, symbol [28] denotes comparison between Foggia and Turkey, symbol [34] denotes comparison between Pavia and Lebanon, symbol [35] denotes comparison between Pavia and Poland, symbol [36] denotes comparison between Pavia and Romania, symbol [37] denotes comparison between Pavia and Spain, symbol [38] denotes comparison between Pavia and Turkey, symbol [45] denotes comparison between Lebanon and Poland, symbol [46] denotes comparison between Lebanon and Romania, symbol [47] denotes comparison between Lebanon and Spain, symbol [48] denotes comparison between Lebanon and Turkey, symbol [56] denotes comparison between Poland and Romania, symbol [57] denotes comparison between Poland and Spain, symbol [58] denotes comparison between Poland and Turkey, symbol [67] denotes comparison between Romania and Spain, symbol [68] denotes comparison between Romania and Turkey, symbol [78] denotes comparison between Spain and Turkey.
